# Efficacy of face coverings in reducing transmission of COVID-19: Calculations based on models of droplet capture

**DOI:** 10.1063/5.0047622

**Published:** 2021-04-27

**Authors:** Joshua F. Robinson, Ioatzin Rios de Anda, Fergus J. Moore, Jonathan P. Reid, Richard P. Sear, C. Patrick Royall

**Affiliations:** 1 H. H. Wills Physics Laboratory, University of Bristol, Bristol BS8 1TL, United Kingdom; 2 School of Mathematics, University Walk, University of Bristol, Bristol BS8 1TW, United Kingdom; 3 School of Chemistry, Cantock's Close, University of Bristol, Bristol BS8 1TS, United Kingdom; 4 Department of Physics, University of Surrey, Guildford GU2 7XH, United Kingdom; 5 Gulliver UMR CNRS 7083, ESPCI Paris, Université PSL, 75005 Paris, France; 6 Centre for Nanoscience and Quantum Information, University of Bristol, Bristol BS8 1FD, United Kingdom

## Abstract

In the COVID-19 pandemic, among the more controversial issues is the use of masks and face coverings. Much of the concern boils down to the question—just how effective are face coverings? One means to address this question is to review our understanding of the physical mechanisms by which masks and coverings operate—steric interception, inertial impaction, diffusion, and electrostatic capture. We enquire as to what extent these can be used to predict the efficacy of coverings. We combine the predictions of the models of these mechanisms which exist in the filtration literature and compare the predictions with recent experiments and lattice Boltzmann simulations, and find reasonable agreement with the former and good agreement with the latter. Building on these results, we explore the parameter space for woven cotton fabrics to show that three-layered cloth masks can be constructed with comparable filtration performance to surgical masks under ideal conditions. Reusable cloth masks thus present an environmentally friendly alternative to surgical masks so long as the face seal is adequate enough to minimize leakage.

## I. INTRODUCTION

Face coverings have become a common (though controversial) motif of the global response to the COVID-19 pandemic.^1–4^ At the time of writing, 139 countries have mandated the use of face coverings (or already practiced universal masking) in public spaces such as on public transport, 19 countries mandate coverings on a regional level and a further 17 countries recommend (but do not require) their use.^5^ The World Health Organization has recently reversed their earlier policy on face coverings and now advise that the public wear them and offer some guidance on the essential features of effective coverings.^6^

SARS-CoV-2 is transmitted primarily by the airborne route, i.e., by direct inhalation of aerosolized particles containing virus.^7–15^ Face coverings work to prevent this transmission route by suppressing onwards transmission of the virus on exhalation^16^ (so-called “source control”) or to provide protection to the wearer on inhalation, i.e., as personal protective equipment (PPE). The former is especially important in this pandemic because the majority of cases of transmission seem to occur from asymptomatic or presymptomatic patients.^4,17–26^ Following the emergence of more infectious variants of SARS-CoV-2, some policy makers have mandated the wearing of medical-grade PPE in public spaces.^27^

The literature on face coverings is limited,^2,28^ and there is a great deal of inconsistency and a lack of clarity in the guidance concerning their use. The academic literature is a combination of medical studies (using either live wearers^29–31^ or mannequins^32–34^) retrospective studies,^2,35–38^ epidemiological modelling^2,39–41^ engineering studies (particularly in the filtration literature),^33,34,42–48^ and aerosol science.^4,7,9,18,36,49–51^ Such a complex phenomenon as airborne transmission depends on very many parameters (e.g., air flow, humidity, separation, mask fit). The disparate disciplines which have considered the use of face coverings take wildly differing approaches, and there seems to be a lack of any consistent experimental protocol, and studies typically only address a subset of the parameters upon which transmission depends.

The mechanisms by which droplets^89^ are captured by filters are reasonably well-established.^52^ There are four principle mechanisms by which droplets may be captured by fibers in a covering which concern us here.^43^

• Steric interception—capture neglecting inertia, so a droplet follows stream lines of the air but collides with a fiber due to the size of the droplet.
• Inertial impaction—where inertia is taken into account resulting in the droplet deviating from stream lines and colliding with the fiber.
• Diffusion—diffusion of droplets in the air leads to contact with a fiber.
• Electrostatic capture—Coulombic and/or dipolar attractions between the droplets and fibers pull the droplet into contact. Note that the previous three mechanisms assume no interaction until particle/fiber contact. Studying this mechanism requires knowledge of the charge distribution in the droplets and fibers.

Gravitation can also play a role in droplet capture; however, this is negligible compared to the other mechanisms outlined above.^53^ The filtration literature's focus on these mechanisms was primarily motivated by developing medical grade PPE. However, experimental work during the pandemic has confirmed the potential of household fabrics to effectively filter some virus-bearing particles.^45–48^

Here, we shall primarily focus on those filtration mechanisms pertinent to droplet capture in cloth masks: interception and inertial impaction. We review the literature which addresses these mechanisms and assess experimental measurements of droplet capture by face coverings. We give a technical account of filtration theory in a rigorous fashion by borrowing some ideas from soft matter physics. By clearly articulating its underlying assumptions, we are able to extend the standard theory to begin to treat household fabrics. Our work provides a route through which mask design can be optimized, and further questions of public policy can be explored in future, e.g., the importance of mask fit.

Our model predicts that multilayered masks made from standard household fabrics should provide comparable filtration performance to surgical masks under ideal conditions, though practical mask performance crucially depends on the fit. We conclude that for many three-layered cloth masks capture of droplets larger than ≳3 μm is highly effective. For smaller (0.1 to 3 *μ*m) droplets, the efficacy is dependent on the type of material from which the face covering is comprised, but some materials can achieve excellent protection (≥95%) for ≳1μm droplets, which is comparable to surgical masks.

This paper is organized as follows: in Sec. II, we describe the experiments exploring the material properties of fabrics. Section III is dedicated to theory and simulations for filtration by a single-fiber. Then in Sec. IV, we investigate the filtration properties of fabrics by combining the work of Secs. II and III. We discuss the significance of our findings and conclude in Sec. V.

## II. MATERIAL PROPERTIES OF MASKS

Fabrics are broadly categorized as knitted, woven, or nonwoven. We refer to face coverings that would be worn by members of the public, that are neither surgical masks nor respirators, as cloth masks, and we use masks as a catch-all term for all kinds of filters. Filtration theory is well-developed for nonwoven materials,^43^ which are typical of surgical masks and respirators. However, cloth masks typically contain knitted or woven fabrics so we introduce some fundamental characteristics of these fabrics below.

**FIG. 1.**
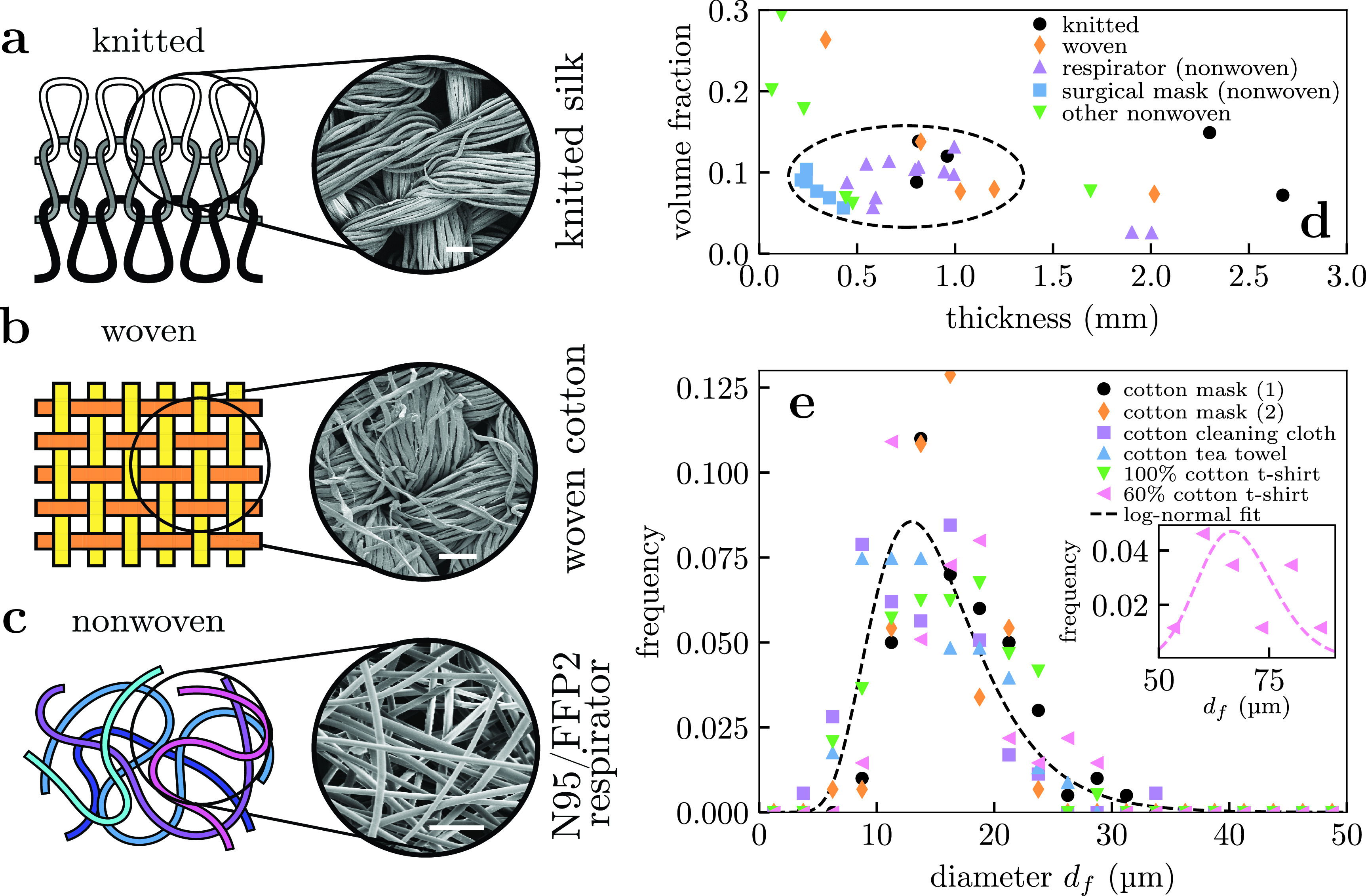
Summary of fabrics comprising masks considered here. (a) Knitted fabrics formed by looping yarn through previous layers (layers colored differently for clarity). (b) Woven fabrics formed by intersecting perpendicular yarns (the “warp” and “weft”). (c) Nonwoven fabrics are formed by entangling fibers through other means, resulting in less ordered arrangements. Scanning electron microscope images of example fabrics in figures (a)–(c) share a scale bar of 100 *μ*m. (d) Geometric properties measured for sample fabric layers, with region of interest marked with a dashed circle (discussed in text). Respirators and surgical masks are comprised of multiple layers, with individual layers plotted separately within this panel. (e) Distribution of fiber diameters in cotton fabric samples, which loosely follow a lognormal distribution. Inset: the 60% cotton 40% polyester t-shirt shows a second peak at larger fiber diameter corresponding to the second material, which can also be modeled as a lognormal (pink dashed).

Knitted and woven fabrics are created by spinning fibers into yarn.^54^ In practice, many of these threads are typically twisted together (the “ply”) into a composite yarn with additional stability against being unwound. Note that the process described above is for staple yarn, where the natural fibers are short, but a different process (filament yarn) may be used where the fibers are naturally long (e.g., silk or synthetic polymers), which results in smoother thread [cf. silk strands are smooth in Fig. 1(a) whereas cotton thread in Fig. 1(b) features stray strands resembling a frayed rope].

Weaving involves interlacing multiple parallel yarn into a tight pattern, whereas knit fabrics are formed by drawing the yarn in complex loops (the “stitches”). Knitting thus results in regions of high curvature, so threads are able to bend which typically results in stretchier fabrics. By contrast, nonwoven materials are formed by entangling the fibers mechanically, thermally, or chemically, which results in a less ordered structure.

The filtration characteristics of masks depends on many parameters, including the size and charge on the droplets as well as mask properties such as fiber thickness, density of fibers, their material composition, and thickness of the mask. In addition, in cloth fabrics details of yarn structure and weave/knit pattern matter. Treating all of these within a single framework represents a significant challenge, so we focus on the most relevant parameters.

### A. Contact forces

All combinations of fibers and droplets interact on contact between the droplet and the fiber, even when they are electrically neutral. In almost all cases we expect droplets to stick when they contact the surface of the fiber. Whether a droplet sticks and spreads on a surface it contacts, or carries on moving, is controlled by the ratios of two competing energies. The first energy acts to keep droplets moving without sticking: the inertial or kinetic energy. The second energy drives sticking and spreading of the droplets: the surface free energy.

For droplets in the size range of interest, the surface free energy is much larger than the kinetic energy, so the surface free energy will win and the droplet will stick—at least in the vast majority of cases. The ratio of the kinetic energy to the surface free energy is the Weber number

$$\text{We}=\frac{\text{kinetic}\;\text{energy}}{\text{surface}\;\text{free}\;\text{energy}}=\frac{\rho_{p}d_{p}U_{0}^{2}}{\gamma },$$

for a droplet of mass density *ρ_p_*, diameter *d_p_*, surface tension *γ*, and moving at speed *U*_0_.

For mucus droplets, $\gamma ∼0.05\;Nm^{-1}$.^55^ For a droplet of diameter $d_{p}∼10\;\mu m$ traveling at 0.1 ms^−1^, $\text{We}∼2\times 10^{-3}$; surface tension forces are then about 500 times stronger than inertial forces, so we expect them to dominate and the vast majority of droplets to stick on contact. Natural fibers such as cotton are more hydrophilic than synthetic polymers used in medical-grade surgical masks and respirators. However, at these very small Weber numbers we do not expect this variation to have a significant effect. Small droplets can even stick to hydrophobic surfaces.^56^

### B. Experiments

We examined a variety of fabrics used to make masks including cloth masks, surgical masks, and respirators. These masks are typically multilayered structures and were decomposed into their individual layers for examination. Their properties are summarized in Fig. 1(d) and a full breakdown is given in Table S1 in the supplementary material.

An important quantity for filtration is the volume fraction of fibers *α*, which we determined from

$$\alpha =\frac{\rho_{A}}{\rho_{b}L},$$ (1)

where *ρ_A_* is the areal density (the “fabric weight,” typically measured in gm^−2^), *ρ_b_* is the bulk density of the fiber, and *L* is the fabric thickness. $\rho_{A}/L$ gives the fabric density. We measured *ρ_A_* by weighing strips of known area and *ρ_b_* is determined from the fabric material (e.g., 1.54 kg m^−3^ for cotton). We measured the fabric thickness by cutting the material into thin strips, clamping them at one end, and measured their thicknesses under bright-field microscopy (Leica DMI 3000B) with a 4× and 10× objective (depending on the thickness of the fabric). This method likely overestimates the thickness for fabrics with a yarn structure: an alternative method for inferring the fabric thickness will be introduced in Sec. IV D (and a comparison of both methods is given in supplementary material). The manufacturers did not state the material composition of the surgical masks and respirators we sampled, so we assumed they were made from polypropylene fibers ($\rho_{b}=0.91\;\text{kg}\;m^{-3}$). We neglect any porosity within the fiber in (1); the scanning electron microscopy (SEM) images in Figs. 1(a)–1(c) and the supplementary material suggests that the porosity is not large enough to significantly affect the measured volume fractions.

We found that the majority of fabric layers were 0.4 to 1.2 mm thick consistent with e.g., Ref. 57 and had volume fractions in the range 0.05≲α≲0.15; these ranges are circled in Fig. 1(d). Notable exceptions to the latter rule included a silk tie and a paper bag with α∼0.26 0.20, respectively; however, we found these samples to be difficult to breath through when placed to the face, making them unsuitable as potential mask materials.

For scanning electron microscopy (SEM) characterization, samples were mounted on SEM stubs and coated with gold/palladium in an Emitech K575X Sputter coater before being imaged in an FEI Quanta 200 FEGSEM (Thermo Fisher Scientific). SEM images were taken at 8 kV using comparable magnifications for all the fabrics. From these images, we manually measured the distribution of fiber diameters *d_f_*, using the open-source software Fiji,^58^ and parameterized it with a lognormal fit. Natural fibers (e.g., cotton) do not have perfectly circular cross sections, so modeling them as cylinders is an approximation. Our measured distribution of fiber diameters will thus be affected by fiber orientation, a consequence of obtaining 3d information from 2d images. A minimum of 50 individual fibers were measured per fabric. The size distributions obtained for cotton samples in Fig. 1(e), and the remaining distributions are given in the supplementary material. For cotton, we find $\text{ln}\;(d_{f}/\mu m)∼N(\mu =2.68,\sigma^{2}=0.12)$, so a cotton layer ∼1mm thick will typically be 50 to 100 fibers thick.

## III. CAPTURE OF DROPLETS BY A SINGLE FIBRE

In this and Sec. IV, we describe the standard theory for filtration of droplets/particles, test its assumptions, and generalize it to incorporate the polydisperse fiber size distributions obtained in Sec. II. In this section, we explore how a single fiber can collect droplets, and in Sec. IV we look at filtration by a fabric formed from a mesh of such fibers. We mostly follow Ref. 43, but we also make use of Refs. 49 and 53. We use the subscript *f* for fiber and *p* for incident particles, e.g., *d_p_* is the particle diameter whereas *d_f_* is the fiber diameter.

### A. Single-fiber efficiency from idealized flows

To understand the filtering capacity of a single fiber, we consider the flow around an infinitely long cylinder aligned perpendicular to the direction of flow. Assuming that the particles faithfully follow the streamlines infinitely far from the cylinder, we define the single-fiber efficiency as the fraction of particles collected by the fiber, i.e.,

$$\eta =\frac{\text{number}\;\text{of}\;\text{collection}\;\text{trajectories}}{\text{number}\;\text{of}\;\text{streamlines}}.$$ (2)

**FIG. 2.**
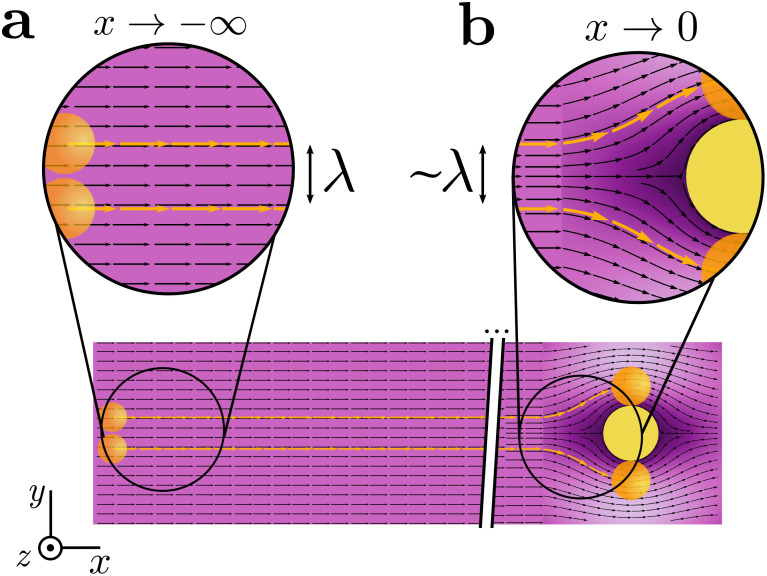
Illustration of single-fiber filtration. Particles moving along trajectories between the upper and lower orange lines collide with the fiber and are filtered out. Particles along these trajectories just glance the surface of a fiber. The width of the collection window, *λ* is defined as being the distance between the upper and lower trajectories far from the fiber, illustrated in (a). Far from the fiber we assume that particles follow the air streamlines. (b) Near the fiber, particle trajectories are highly curved precluding a simple geometric interpretation of *λ*. *λ* depends on the particle and fiber sizes, as well as the background gas flow. Lighter (darker) shading corresponds to faster (slower) background flow speed.

Infinitely far from the mask, the velocity field is $u=U_{0}e_{x}$ so that the streamlines are distributed uniformly on planes with normal vector $e_{x}$, as in Fig. 2(a). We assumed *z*-symmetry so that our problem geometry is two-dimensional in the *xy*-plane, so this leaves width (in the *y*-direction) as a suitable measure for the number of streamlines. Given these considerations, we can write the single fiber efficiency as $\eta =\lambda /L_{y}$ where *λ* is the width of the collection window in Fig. 2 and *L_y_* is the total width of the mask in the *y*-direction.

Our definition of single-fiber efficiency differs from that normally used in filtration literature, namely the quantity $\lambda /d_{f}$ in, e.g., Refs. 43, 49, and 53. We have chosen a definition which guarantees η<1 so it can be interpreted as a probability; the more common definition is not properly normalized, which can lead to incorrect and poorly posed results when combining multiple collection mechanisms (cf. Sec. III A 5).

#### 1. Kuwabara flow field

Flow through a filter occurs at low Reynolds number, so it is well described by Stokes flow. There is no solution to Stokes flow around a free cylinder because of the Stokes' paradox;,^59^ however, the mask is composed of many fibers and we can obtain a solution for flow around a fiber immersed in an effective neighborhood of other fibers: the Kuwabara flow.^60^ The effective neighborhood is treated as an outer circle boundary at distance $a_{f}/\sqrt{\alpha }$ where *a_f_* is the radius of the fiber, so that the flow is modeled in the coaxial region $a_{f}\leq \rho \leq a_{f}/\sqrt{\alpha }$ which allows solution without a paradox. Moreover, the radial component of the velocity at the outer boundary is taken as $u_{\rho }(\rho =a_{f}/\sqrt{\alpha })=U_{0}\;\text{cos}\;\theta$. *U*_0_ is the average flow speed through the mask, obtained from the flow speed at the mask surface (cf. Table I).

**TABLE I.** Key parameter values for masks including air, water, and mucus at 20 aC and atmospheric pressure 10^5^ Pa. Note that small droplets dry rapidly and this will cause their viscosity to increase. Flow rates are determined from the volume typically exhaled during one minute. Moderate exertion is defined as that readily able to be sustained daily during 8 h of work, whereas maximal exertion is the upper limit of what can be sustained for short periods of time (e.g., during competitive sports). Flow speeds are calculated for the stated mask area and flow rates assuming perfect face seal; in practice, leakage would reduce flow through the mask.

Quantity	Value	Reference
Air		
Mass density	1.2 kg m^−3^	68
Dynamic viscosity *μ*	1.8 × 10^−5^ Pa s	68
Kinematic viscosity *ν*	1.5 × 10^−5^ m^2^ s^−1^	68
Water/mucus		
Mass density *ρ_p_* (water)	998 kg m^−1^	68
Dynamic viscosity (mucus)	0.1 Pa s	55
Mucus/air surface tension *γ*	$0.05\;Nm^{-1}$	55
Typical breathing flow rates		
Tidal breathing at rest	6 l min^−1^	69
During mild exertion	20 l min^−1^	69
During moderate exertion	30 l min^−1^	69
During maximal exertion	85 l min^−1^	69
Average flow speeds		
Effective mask area	190 cm^2^	70
Flow speed (rest)	0.5 cm s^−1^	
Flow speed (mild)	1.8 cm s^−1^	
Flow speed (moderate)	2.7 cm s^−1^	
Flow speed (maximal)	7.5 cm s^−1^	

For incompressible flow ∇·u=0 the velocity field can be expressed in terms of a streamfunction, i.e.,

u=∇×ψ, (3)

where

$$\psi (\rho ,\theta )=U_{0}f(\rho )\;\text{sin}\;\theta \;e_{x},$$ (4a)

$$f(\rho )=\frac{f_{1}}{\rho }+f_{2}\rho +f_{3}\rho^{3}+f_{4}\rho \;\text{ln}\;\left(\frac{\rho }{a_{f}}\right),$$ (4b)

with coefficients $\left\{f_{i}\right\}$ set by the boundary conditions. The Kuwabara flow field is obtained by assuming the velocity vanishes on the fiber surface $u(\rho =a_{f})=0$, and that the vorticity ∇×u vanishes at the outer boundary $\rho =a_{f}/\sqrt{\alpha }$ to approximate the neighborhood around the fiber.^60^ We give the explicit values of the coefficients obtained in the supplementary material.

#### 2. Lattice Boltzmann flow field

To test the validity of the Kuwabara flow field, we also calculated flow fields using Lattice Boltzmann (LB) simulations.^61–64^ In these simulations, the Reynolds number Re is nonzero and can be varied, and the fluid is compressible. However, at our small Re the spatial variation in density is very small. To do the LB simulations, we use a modified version of a code from PALABOS group at the University of Geneva.^65^ See the supplementary material for details.

We have performed two types of LB simulations. In the first we can calculate the flow field around a single fiber, which allows us to calculate the single-fiber collection window *λ*. In the second we calculate the flow field in a disordered hexagonal lattice of fibers, which is our model of a mask. This flow field allows us to test the theory's ability to predict filtration efficiency, at least within our simple two-dimensional model. In all cases, we run the LB simulations until we reach steady state and then use the steady-state flow field.

#### 3. Particle motion

The equation for particle velocity **v** (Newton's second law) while being transported by the flow **u** is

$$m_{p}\frac{dv}{dt}=-\frac{v-u}{B}+F,$$ (5)

where *m_p_* is its mass. The first term on the right-hand side is the Stokes drag. In this term $B=C/6\pi \mu a_{p}$ is the particle mobility, with *μ* the dynamic viscosity of air and *C* the Cunningham slip correction factor.^66,67^ **F** contains any other external forces such as gravity, which we do not consider here. We have assumed that the particle interacts with the flow field as a point particle so that (a) the flow field **u** is unperturbed by the presence of the particle and (b) the Stokes drag couples only to the particle's center of mass.

We denote dimensionless parameters with tildes, defined through the transformations $u=U_{0}\tilde{u},\;v=U_{0}\tilde{v},\;r=a_{f}\tilde{r}$, and $t=a_{f}\tilde{t}/U_{0}$ so (5) becomes^90^

$$\text{St}\frac{d\tilde{v}}{d\tilde{t}}=-(\tilde{v}-\tilde{u})+\frac{B}{U_{0}}F,$$ (6)

with Stokes number

$$\text{St}=\frac{m_{p}U_{0}B}{a_{f}}=\frac{2\rho_{p}a_{p}^{2}U_{0}C}{9\mu a_{f}}∼\frac{6.2\times 10^{6}}{m^{2}s^{-1}}\frac{d_{p}^{2}}{d_{f}}U_{0}C,$$ (7)

with the latter step evaluated for parameter values typical of incoming droplets. These are in Table I. The Stokes number describes the effective inertia of the particle moving under the flow field. For threads with diameter O(100μm), typical of yarns used in knitted and woven fabrics, we find St≪1 making inertia irrelevant for particles around O(1μm) in diameter; for this reason, the smaller fibers are crucial for capture of exhaled droplets in cloth masks.

#### 4. Particle deposition and collection efficiency

For the LB flow field, the length of the single-fiber collection window *λ* can be determined by direct measurement of its geometric definition in Fig. 2. The Kuwabara flow field is only valid in the region of high curvature close to the fiber surface, so determining *λ* is slightly more subtle.

**FIG. 3.**
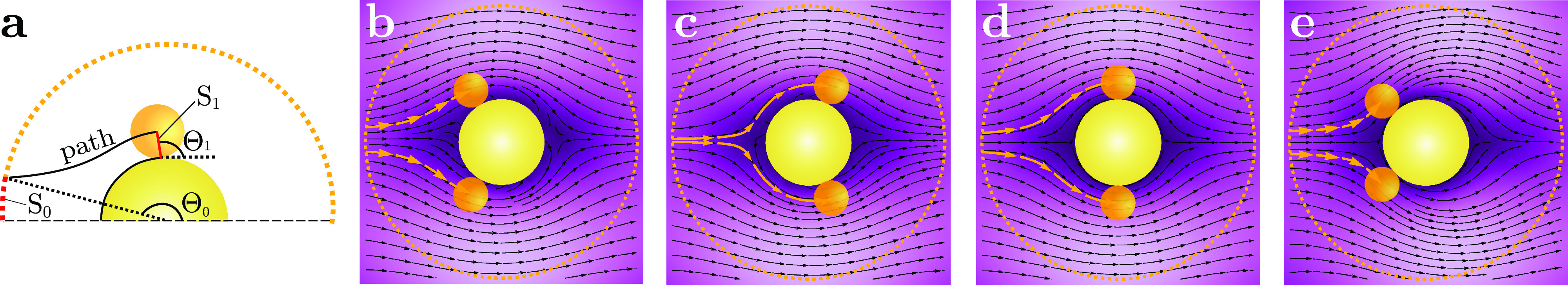
Geometry of particle capture in the Kuwabara flow field. Lighter (darker) shading corresponds to faster (slower) flow speed. (a) Diagram of limiting trajectory: the particle path which only just collides with the fiber. In the absence of attractive forces and inertia the capture angle will be $\theta_{1}=\pi /2$. (b) and (c) Effect of spherically symmetric forces on the incoming particle trajectories. The forces move the limiting trajectory toward the near or far side of the fiber depending on whether the interaction is attractive (b) or repulsive (c). (d) and (e) Inertia brings the limiting trajectories toward the near side of the collecting fiber, shown are particle trajectories for (d) St=0 and (e) St=0.5.

Defining *n* as the number density of incoming particles, the continuity equation in the steady-state $\dot{n}=0$ yields ∇·(nv)=0. All particle trajectories that terminate on the fiber surface are contained in the volume bounded by the limiting path shown by a solid black line in Fig. 3(a). We integrate the continuity equation over this and apply the divergence theorem to give

$$\int_{S_{0}}nv\cdot dS+\int_{S_{1}}nv\cdot dS=0,$$ (8)

using the fact that the v·dS=0 along the limiting trajectory and the fiber surface at *r* = *a_f_*, and the surfaces $S_{\left\{0,1\right\}}$ are defined in Fig. 3(a). We write the magnitude of either integral in the above expression as Φ/2: (half) the rate of particle deposition on the fiber surface. We multiply by two to account for collection along both sides of the fiber, taking advantage of the symmetry in the *y*-direction.

The width of the collection window is determined from the deposition rate by $\lambda =\Phi /n_{0}U_{0}L_{z}$ where *n*_0_ is the particle number density far away from the fiber and *U*_0_ is the flow speed. We apply the boundary condition $n(r=a_{f}/\sqrt{\alpha })=n_{0}$, which is a constant along *S*_0_, so we have the following expression for collection efficiency:

$$\lambda =\frac{d_{f}}{\sqrt{\alpha }}\int_{\pi }^{\theta_{0}}{\tilde{v}}_{\rho }\left(\theta ;\rho =\frac{a_{f}}{\sqrt{\alpha }}\right)\;d\theta .$$ (9)

The velocity field at the outer boundary is a boundary condition of the field, so *θ*_0_ is the key quantity needed to evaluate efficiency through this route. For v=u at the boundary this reduces to

$$\lambda =d_{f}\;\text{sin}\;(\theta_{0})f\left(\frac{a_{f}}{\sqrt{\alpha }}\right).$$

The angle *θ*_0_ is obtained by following the limiting trajectory [e.g., the one shown in Fig. 3(a)] that only just glances the fiber. Particle trajectories in this limit are defined by

$$\frac{1}{\rho }\frac{d\rho }{d\theta }=\frac{v_{\rho }}{v_{\theta }}=\frac{u_{\rho }}{u_{\theta }},$$ (10)

which can be integrated backwards in time with final conditions *r* = *a_f_* and $\theta =\theta_{1}=\pi /2$ to determine *θ*_0_.

#### 5. Single-fiber efficiency from combined mechanisms

From the definition of the single-fiber collection efficiency (2), we can see that if the mechanisms act completely independently then the penetration probability, the probability of passing the fiber will be the product of the penetration probabilities due to the individual mechanisms, i.e.,

$$1-\eta =\prod_{k}\left(1-\frac{\lambda_{k}}{L_{y}}\right)=1-\sum_{k}\frac{\lambda_{k}}{L_{y}}+O\left({\left(\frac{\lambda_{k}}{L_{y}}\right)}^{2}\right),$$

where *k* sums over the different mechanisms and the last step is valid in the macroscopic limit ${\left(\lambda /L_{y}\right)}^{2}\ll 1$. However, in practice these mechanisms are not independent and the relative catchment lengths *λ_k_* will overlap. Assuming perfect overlap and no interaction between mechanisms, the total efficiency will simply equal the most efficient individual mechanism, i.e., $\text{max}(\{\eta_{k}\})$.

Combining the two limits above, we find

$$\frac{\text{max}(\{\lambda_{k}\})}{L_{y}}\leq \eta \leq \sum_{k}\frac{\lambda_{k}}{L_{y}}.$$

If one mechanism dominates over the others, then these two bounds converge and we can simply take the dominant mechanism.

#### 6. Specific mechanisms

As noted in the introduction, there are four principle mechanisms by which droplets may be captured by a mask which concern us here, steric interception, inertial impaction, diffusion, and electrostatic capture.^43^ These mechanisms generally act in different size regimes, so it is simpler to calculate their effects in isolation and then combine them using the approach outlined in Sec. III A 5. The SARS-CoV-2 virus is ∼0.1μm in diameter, so this is the smallest size of interest. Exhaled droplets have been observed across the wide range of O(0.1−100μm), which corresponds to Stokes numbers from 10^−4^ to 10^3^. However, the majority of droplets are larger than ≥1μm^71,72^ where $\text{St}≳10^{-3}-10^{-2}$, and coarser droplets are expected to contain more virus on average.^73,74^ The ≥1μm size regime is therefore of most interest, and the importance of the finer regime O(0.1−1μm) will be scenario-dependent.

Electrostatic capture is crucial for high efficiency filtration of particles with size of order O(0.1μm) in respirators which make use of electret fibers that sustain surface charges *σ*_0_ of order $O(1{\text{nCcm}}^{-2})$.^53,75,91^ The electrostatic forces in electrets are typically an order of magnitude more efficient at capture than the mechanical forces, and this efficiency is expected to scale as $\propto \sigma_{0}$ for the Coulombic force or $\propto \sigma_{0}^{2}$ for the dielectrophoretic force.^53^ However, the surface charge density is typically two orders of magnitude smaller in cloth masks^92^ and so electrostatic capture should be an order of magnitude less efficient than for the first three mechanical mechanisms. We therefore neglect electrostatic capture in this work.

For interception, collection occurs when the finite-sized particles touch the surface of the fiber while passing, with the limiting trajectory occurring at $\theta_{1}=\pi /2$. The particle follows the flow v=u (inertia is included in *impaction* but not in interception) and the limiting trajectory occurs at $\theta_{1}=\pi /2$, so (9) gives

$$\lambda_{R}=\frac{2\psi (a_{f}+a_{p},\pi /2)}{U_{0}}.$$ (11)

In general, capture efficiency is further enhanced by diffusion and inertia. The role of diffusion is quantified by the Péclet number,

$$\text{Pe}=\frac{\text{rate}\;\text{of}\;\text{convection}}{\text{rate}\;\text{of}\;\text{diffusion}}=\frac{d_{f}U_{0}}{D},$$ (12)

where *D* is the particle diffusion coefficient for motion relative to the flow. We find that Pe≪1 for $d_{p}≳1\mu m$ so diffusion is negligible for capture of larger droplets. Similarly, inertia plays no role in the capture of smaller droplets $d_{p}≲0.1\mu m$ because St≪1 in that regime. Most exhaled droplets are larger than $d_{p}≳1\mu m$;^71,72^ thus, inertia is crucial to the effectiveness of cloth masks in the relevant size regime and warrants a more detailed treatment. We use standard results for diffusion, given in the supplementary material.

To determine the single-fiber collection window *λ* for finite Stokes number St, we use an iterative scheme where we test whether a particular initial angle leads to collision with the fiber, and update a lower and upper bound for *θ*_0_ accordingly. By testing for collision for the midpoint between the current bounds, we ensure each iteration adds ∼1 bit of information to the approximation of *λ* and convergence is rapid. For the LB flow field, we use a similar scheme, but varying the initial height of the particle far from the mask where the flow is parallel (cf. Fig. 2).

### B. Droplet inertia rapidly increases efficiency above a threshold value

Inertia causes droplets to deviate from streamlines which can bring particles closer to the fiber enhancing capture. The inertia, as measured by the Stokes' number St in (7), increases as $d_{p}^{2}$ so this mode dominates capture of large droplets. Naively, we would expect this increase in efficiency to be a simple increasing function of the Stokes number. However, inertia also carries particles closer to the fiber where the flow is slower and more curved, which increases the opposing forces acting against the particle; this creates competition and inertial capture is nontrivial for intermediate values of St.

**FIG. 4.**
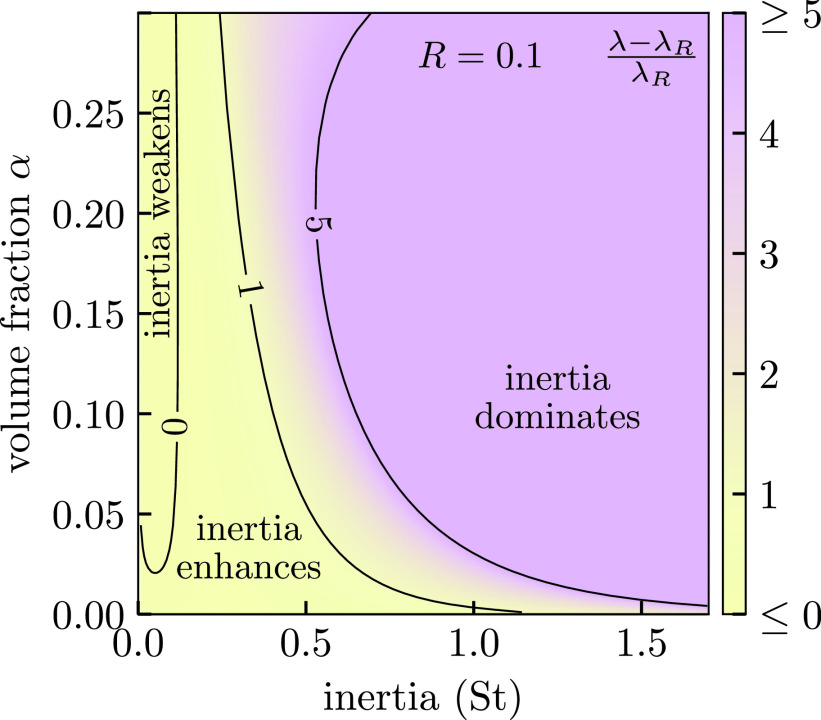
Deviation $\lambda /\lambda_{R}-1$ of single-fiber collection efficiency *λ* from the interception capture efficiency *λ_R_* for finite particle-to-fiber size ratio $R=d_{p}/d_{f}=0.1$. We see a sharp crossover from interception to inertial capture as the dominant mechanism. *λ* increases by a factor of ≳5 as St is increased to ∼0.5. *λ_R_* is defined in (11). We assumed the particle moves in the Kuwabara flow field in these calculations.

In Fig. 4, we show how *λ* varies with *α* and St. There is a sharp crossover from weak to strong capture as St reaches values in the O(0.1) range when α≳0.01. This sharp crossover is a residual of an underlying dynamical transition occurring in the point particle limit $d_{p}/d_{f}\to 0$ demonstrated by Araújo, Andrade, and Herrmann.^77^ We will explore this transition in more detail in a future manuscript, but here the important message is that once inertia becomes a relevant mechanism the total mask efficiency will rapidly increase (with particle size) to unity independent of the mask details. However, the location of this crossover does depend on the mask properties. Curiously, we find that for small St there is a region where inertia decreases the efficiency of capture for finite *R* highlighting that capture efficiency has a nontrivial dependence on inertia.

**FIG. 5.**
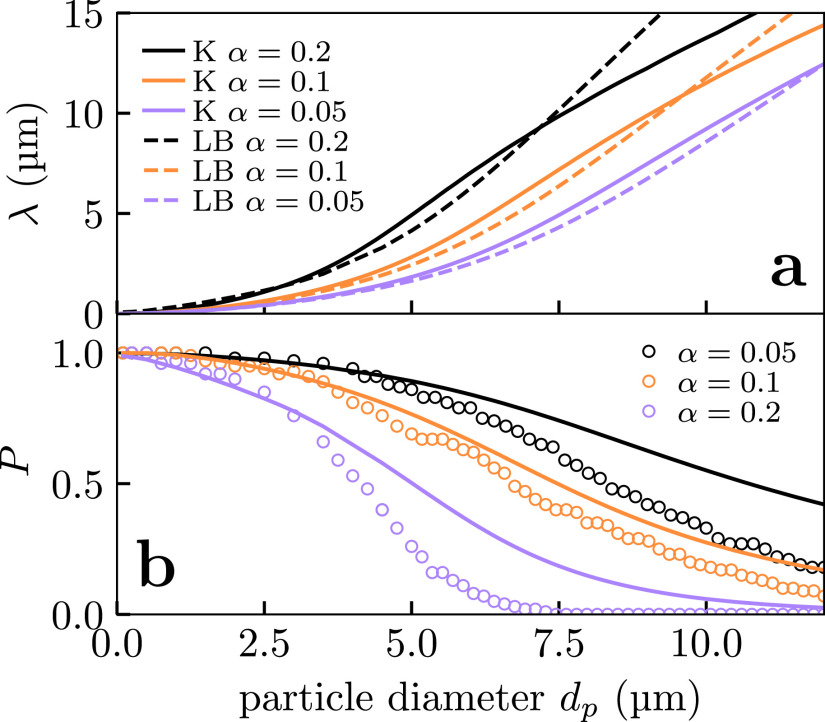
Comparison of theoretical model against lattice Boltzmann simulations. (a) Plot of the single fiber *λ* as a function of particle diameter calculated from the Kuwabara (solid lines) and LB (dashed lines) flow fields. (b) Comparison between the penetration *P* calculated using LB simulations of model filters (points) with the predictions of (13a) (curves). In both cases, the flow speed $U_{0}=2.7\;\text{cm}\;s{\;}^{-1}$ and the fiber diameter $d_{f}=15\;\mu m$ with α=0.05, 0.1, and 0.2.

All the above calculations used the approximate Kuwabara flow field to compute *λ*. We performed LB simulations to check the validity of the Kuwabara approximation. Kuwabara and LB values for *λ* are compared in Fig. 5(a). We note that, especially at small fiber volume fraction *α*, the Kuwabara approximation gives *λ* values close to those obtained by LB simulations. So we conclude that at least under most conditions, the Kuwabara flow field yields good approximations for *λ*.

Above the dynamical transition, *λ* increases rapidly with particle size, see Fig. 5(a), due to the effect of increasing inertia. So in this regime, typically of particles micrometers in diameter, the filtration efficiency increases rapidly. To see this, consider a fiber of diameter 15μm [typical of cotton from Fig. 1(e)], in air for a flow speed of $2.7\;\text{cm}\;s{\;}^{-1}$ corresponding to breathing during moderate exertion. LB calculations for a particle of diameter 2 μm find a collection range λ=0.36 μm or about 2.5% of the fiber width. However, increasing the particle diameter to 8 μm yields a collection range λ=7.1 μm or almost half the fiber width.

## IV. FROM SINGLE FIBRES TO TOTAL FILTER EFFICIENCY

In Sec. III, we developed the theory for the width of the region over which a single fiber collects the droplets: *λ*. In this section, we model a filter as an array of these fibers and calculate filtration efficiencies from *λ*, the volume fraction *α,* and thickness of the filter. Standard filtration theory assumes the fibers are identical in shape and size, act (i.e., filter) independently and are distributed homogeneously in space. These assumptions are reasonable for nonwoven materials such as surgical masks; however, in common fabrics we typically find

1. The individual fibers vary in shape and size.
2. In woven and knitted fabrics, the fibers are hierarchically arranged because of the yarn structure. The fibers are densely packed in yarns, leaving regions of lower density in the inter-yarn pores.

Our treatment generalizes filtration theory to account for these heterogeneities. We present these generalizations in Secs. IV A–IV D and numerically compare the resulting theory against the experimental data available from the literature.

### A. Filter efficiency from a polydisperse assembly of fibers

Standard filtration theory considers filters as an assembly of identical cylindrical fibers. Here, we borrow ideas from statistical mechanics to rigorously formulate the main result of filtration theory, as well as provide the natural generalization for when the fibers vary in diameter. As we noted in Sec. II B, natural fibers are seldom perfectly cylindrical so this formulation is approximate.

For simplicity, we consider a rectangular filter of dimensions $\left(L_{x},L_{y},L_{z}\right)$, although the shape details perpendicular to the direction of flow do not matter because we will ultimately consider the limit of an infinite plane. On average, the streamlines (carrying particles) will occupy an effective area of (1−α)A, so the effective efficiency is modified to $\eta_{k}=\lambda_{k}/((1-\alpha )L_{y})$, where we have introduced a subscript *k* for the efficiency of fiber *k* as materials are generally heterogeneous and *λ* will be taken from a distribution of values [cf. distribution of fiber sizes in Fig. 1(e)]. Assuming the results for single fibers of Sec. III, the probability that a particle is collected by fiber *k* then equals the probability that a cylinder of diameter *λ_k_* crosses the particle path. Those results assume that all the fibers are aligned perpendicular to the flow direction.

In the simplest case where the particle trajectory is a straight line through the filter, the probability that a particle passes the *k*th fiber is $P_{k}^{\left(1\right)}=1-\eta_{k}$. Assuming the fibers act independently gives the penetration, the total fraction of particles that pass through the filter, as

$$P=\underset{L_{y}\to \infty }{\lim}\prod_{k=1}^{N}P_{k}^{\left(1\right)},$$

where $N=nL_{x}L_{y}$ is the total number of fibers in terms of fiber density (number per unit cross-sectional area) $n=4\alpha /\pi d_{f}^{2}$. Geometrically, the $L_{y}\to \infty$ limit above takes the limiting geometry as an infinite plate (as $L_{z}\to \infty$ is already implicit in our 2d formulation). We take this limit by considering the logarithm of both sides, giving

$$\text{ln}\;P=\underset{L_{y}\to \infty }{\lim}nL_{x}L_{y}\int_{{\mathbb{R}}^{+}}\;\text{ln}\;p(d_{f})\;d\mu (d_{f}),$$

which introduces the measure on the fiber size distribution $\mu (d_{f})$ that is normalized through $\int_{{\mathbb{R}}^{+}}d\mu (d_{f})=1$. Taking the limit yields

$$\underset{L_{y}\to \infty }{\lim}L_{y}\;\text{ln}\;\left(1-\frac{\lambda }{(1-\alpha )L_{y}}\right)=-\frac{\lambda }{1-\alpha },$$

so the total penetration becomes

(13) $$P=\text{exp}\;\left(-\frac{L_{x}}{\xi }\right),$$ (13a)

with penetration length

$$\xi =\frac{(1-\alpha )\pi }{4\alpha \bar{\lambda }}\int_{{\mathbb{R}}^{+}}d_{f}^{2}\;d\mu (d_{f}),$$ (13b)

and effective collection window

$$\bar{\lambda }=\int_{{\mathbb{R}}^{+}}\lambda (d_{f})\;d\mu (d_{f}).$$ (13c)

Finally, we take the measure to be a lognormal distribution based on the fits to the experimental measurements described in Sec. II B (cf. Table S1 in supplementary material).

Our fundamental assumptions to achieve the above expressions were that (a) the fibers act independently, and (b) their sizes are independent and identically distributed random variables. We directly test assumption (a) in Sec. II D of the supplementary material.

In Fig. 5(b), we compare the predictions of (13) with the penetrations observed in LB simulations of a disordered lattice of fibers. We see that (13) systematically overpredicts the penetration, but that the error is typically relatively small. Thus, as the model is only a very simplified realization of a mask, we conclude that the approximations involved in (13) give an acceptable level of accuracy. Note that due to the Stokes' paradox,^59^ fibers are never completely independent of each other. Moreover, fibers will be arranged in a disordered fashion and so there will be variation in the distances between neighboring fibers, so (13) essentially both neglects correlations and assumes each fiber has the same local environment.

### B. Filtration efficiency of nonwoven materials

The theory of Sec. IV A is sufficient to predict the filtration efficiency of nonwoven materials. To demonstrate this, we compare the predictions of our model against experimental data for three surgical masks from Refs. 45 and 46 (SM1, SM2, and SM3). The physical properties of these masks were not stated, so for comparison we sampled two new surgical masks (SM4 and SM5) and characterized their thickness and fiber distribution using the methods in Sec. II B. These surgical masks consisted of three layers with distinct properties and thus penetrations through individual application of (13). Equation (13) implies that layers act independently, so the total mask penetration was obtained by combining the penetrations of the individual layers multiplicatively.

**FIG. 6.**
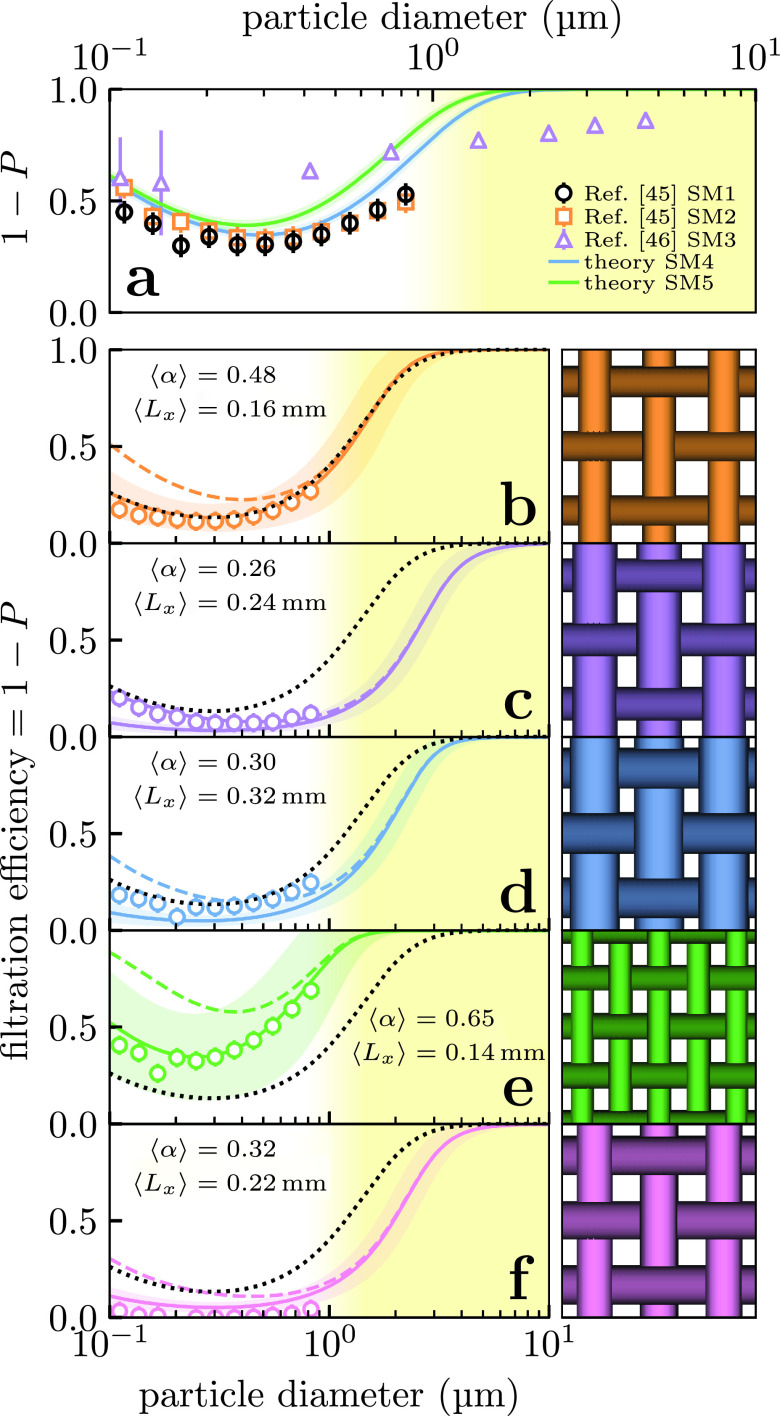
Comparison between our theoretical model (lines) and the experimentally determined filtration efficiencies (points) of Refs. 45 and 46 for (a) surgical masks and (b)–(f) the plain-woven cotton fabrics considered in Ref. 45 (numbered 1–4 and 11 there and in Table II). The filled region surrounding the theoretical prediction indicates the confidence interval from propagating the uncertainties in the experimentally determined parameters. For reference, the left panels in (b)–(f) show our “zeroth-order” prediction where we ignore the inter-yarn pores (dashed) and 1/3 of surgical mask SM4 (black dotted). The right panels in (b)–(f) are illustrations of 1 mm^2^ square regions of each fabric.

Our results compare favorably against the literature data in Fig. 6(a). Our theoretical prediction for these masks closely matches the precise data of Ref. 45 for their own masks (SM1 and SM2). Our theory captures the experimental behavior without any free parameters. Moreover, our model agrees with the trend of increasing filtration efficiency going into the micrometer regime seen in Ref. 46 (SM3). There was a small amount of variation in the physical properties we observed in masks SM4 and SM5 (parameters given in supplementary material) which creates some variation in filtration efficiency. The small deviations from the precise data of Ref. 45 may therefore arise from differences in mask manufacture.

### C. Ease of breathing through a mask and the effect of hierarchical structure on the flow

The pressure drop across a homogeneous filter Δp is given by^43^

$$\Delta p=\frac{\mu L_{x}U_{0}f_{p}(\alpha )}{d_{f}^{2}},$$ (14)

where the function $f_{p}(\alpha )=16\alpha /K$ for the Kuwabara flow field or it can be estimated from previous empirical studies.^43^ The pressure drop across the mask needed for a given flow speed *U*_0_, scales with this speed as well as mask thickness placing limits on how thick masks can be made. The variation with fiber size as $d_{f}^{-2}$ (which follows directly from Poiseuille flow) makes finer fibers harder to breathe through. This is often expressed in terms of a filter quality factor *q* such that $P=e^{-q\Delta p}$.^43,53^

Pressure drops measured across masks vary from a few Pa (Ref. 46) to 100 Pa and above.^48^ This pressure drop cannot be too large, to allow easy breathing. The N95 standard specifies maximum values for Δp of 343 Pa on inhalation and 245 Pa on exhalation (at flow rates of 85 l min^−1^).^79,80^ With a fixed limit to Δp, there are really only two factors that we can vary: the particle collection efficiency of a single fiber, *λ*, and the mask geometry through *α*. In practice, the quality factor *q* can be optimized by varying the geometric parameters *d_f_* and *α* (and thus implicitly *λ*) by, e.g., combining layers of different materials. The resulting efficiency from combining fabric layers has been explored extensively in experiments in Refs. 46 and 48.

**FIG. 7.**
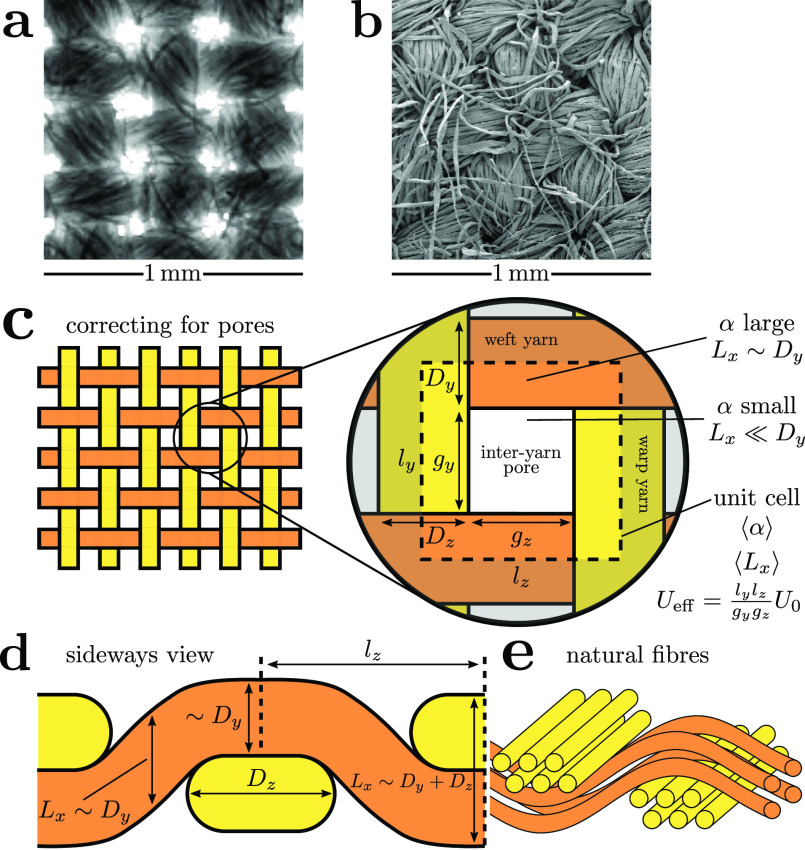
(a) and (b) The same woven cotton layer under (a) optical and (b) scanning electron microscopy. (c) Schematic of how we treat heterogeneous woven fabric as an effective homogeneous medium by averaging over the geometric parameters over the dense yarn and sparse pore regions. (d) Sideways view of a yarn showing the local fabric thicknesses taken for averaging. Elastic deformations flatten the yarns' cross-sections into stadium shapes.^78^ (e) Idealized decomposition of yarns into their constituent fibers.

For spatially heterogeneous masks (woven or knitted) (14) no longer applies. However, from mechanical considerations the pressure drop must be independent of the path through the mask which allows us to treat this more general case. We will consider the effect this has on the flow through woven materials illustrated in Fig. 7. Specifically, we consider the inter-yarn pore regions shown in Figs. 7(a)–7(c). The pores are seen as the light regions under bright-field microscopy in Fig. 7(a); however, SEM [Fig. 7(b)] reveals that these pores are not empty and so droplet capture can still occur there. However, these pores contain considerably fewer fibers than inside the yarns so the flow is faster there.

If *U*_0_ corresponds to the average flow speed through the entire fabric (constrained by the breathing rate), then we generally expect to find $U_{f}\ll U_{0}\ll U_{p}$ where *U_f_* and *U_p_* are respectively the average flow speeds through the dense yarn and sparse inter-yarn pore regions. Typical flow speeds can be estimated by inserting *U_f_* into (14) and equating the pressure drop with that expected through the inter-yarn pores assuming Poiseuille flow. This yields a relationship between *U_f_* and *U_p_* in terms of the pore area fraction

$$\kappa =\frac{g_{y}g_{z}}{l_{y}l_{z}}.$$ (15)

For typical values of *κ* we find that ≳99% of the flow is expected to go through the pore region, and the average flow inside the pore is approximately

$$U_{p}≃\frac{U_{0}}{\kappa }.$$ (16)

This is related to the long-standing “stagnant core problem” of laundry detergency.^81^

### D. Extending filtration to woven and knitted materials

#### 1. Zeroth-order approximation: Ignoring pores

As a zeroth-order approximation to modeling spatially heterogeneous fabrics, we treat them as an effective homogeneous (nonwoven) medium. We assign each fabric an average quantity ⟨α⟩ and $\left\langle L_{x}\right\rangle$, obtained by averaging over the fabric unit cell shown in Fig. 7(a). Figure 7(b) shows how yarns elastically deform to have stadium cross-sections where they interlock,^78^ which we approximate as a rectangular cross-section to simplify the averaging procedure. Thus, the local thickness of the fabric simply equals the sum of diameters of any yarns present while traversing the unit cell in Fig. 7(c); consequently, we take the thickness to be zero in the pore region and assign *L_x_* as in Fig. 7(d) where there are yarns:

• $L_{x}=D_{y}+D_{z}$ in the four corner regions of the unit cell, occupying a total area $D_{y}D_{z}$.
• *L_x_* = *D_y_* or *D_z_* in the rectangular regions where there is a single yarn, with areas $g_{z}D_{y}$ and $g_{y}D_{z}$.

$D_{y},D_{z}\gg d_{f}$ are the thicknesses of the warp and weft yarns (cf. Figure 7), which we obtained for our sample fabrics in Sec. II B and Zangmeister *et al.* state these for their fabrics and summarized in Table II. This gives the average thickness as

$$\langle L_{x}\rangle =\frac{g_{y}D_{z}^{2}+g_{z}D_{y}^{2}+(D_{y}+D_{z})D_{y}D_{z}}{l_{y}l_{z}}.$$ (17)

**TABLE II.** Parameters characterizing the plain-woven cotton fabrics considered in Ref. 45, with estimated last-digit uncertainties given in parentheses. These were estimated from the measurements given in the supplementary material of Ref. 45, together with Eqs. (1) and (17) for $\left\langle L_{x}\right\rangle$ and ⟨α⟩ as described in the text.

Fabric	$D_{x}/\text{mm}$	$D_{y}/\text{mm}$	$g_{x}/\text{mm}$	$g_{y}/\text{mm}$	$\langle L_{x}\rangle /\text{mm}$	⟨α⟩
1	0.17 (1)	0.15 (1)	0.33 (6)	0.33 (6)	0.16 (2)	0.48 (7)
2	0.23 (1)	0.17 (1)	0.33 (6)	0.33 (6)	0.24 (3)	0.26 (4)
3	0.25 (1)	0.21 (1)	0.33 (6)	0.33 (6)	0.32 (4)	0.30 (4)
4	0.12 (1)	0.13 (1)	0.20 (2)	0.25 (3)	0.14 (2)	0.65 (9)
11	0.19 (1)	0.19 (1)	0.33 (6)	0.33 (6)	0.22 (3)	0.32 (4)

The average volume fraction ⟨α⟩ is then obtained from (1) by combining $\left\langle L_{x}\right\rangle$ with the fabric weight and the bulk density of the material. By inserting these spatially averaged parameters into (13), we can treat a woven fabric as an effectively homogeneous (nonwoven) one. We thus assume an average flow of *U*_0_ through this effective medium in this zeroth-order approximation.

We compare this approximation (dashed line) to literature experimental data for several plain-woven cotton fabrics considered in Zangmeister *et al.* in Figs. 6(b)–6(f). The agreement with the literature data are poor for small particles, but improves approaching larger particle sizes of $d_{p}\;∼1\;\mu m$. The smallest particles are unlikely to contain even a single virion, however the poor agreement causes us to overestimate the efficiency in the intermediate size regime so it is worthwhile to improve on this approximation. We consider the sources of disagreement below and attempt to refine the model.

#### 2. Correction for pores

In the Sec. IV C, we found that most of the flow is expected to go through the inter-yarn pores in textiled materials. Consequently, compared to flow through a homogeneous material, (i) the effective fiber density will be reduced and (ii) the typical flow speed will be increased.

Effect (i) generically lowers the collection efficiency as there are fewer fibers to collect particles, whereas the effect of (ii) depends on the collection mechanism. Collection by inertia (impaction) is enhanced by increasing the flow speed, opposing the effect from an effectively reduced fiber density. After cancelation, we thus expect the resulting change in efficiency to be small, and so we do not correct this collection mechanism. However, the efficiency of collection by diffusion decreases with increasing flow speed, reinforcing effect (i), which is potentially significant.

We attempt to correct the efficiency of filtration by diffusion by replacing *U*_0_ with the approximate pore flow speed (16) in our calculated Péclet number (12). We estimate the pore area fraction *κ* using (15) with the yarn parameters given in Ref. 45. When we use this flow speed in the expression for diffusion efficiency (supplementary material IV), we obtain a final filtration efficiency that more closely matches the experimental data of Zangmeister *et al.* in Figs. 6(b)–6(f). While the precise data of Zangmeister *et al.* does not extend into the micrometer regime, the correct position of the minima in Figs. 6(b)–6(f) and the trend toward increasing efficiency approaching 1μm [especially in Fig. 6(e)] indicates that leaving inertia uncorrected is reasonable.

Considerable variation from fabric to fabric was reported in Refs. 45, 46, and 48, some of which is seen in Figs. 6(b)–6(f). For example, the fabric in Fig. 6(b) is roughly equivalent to a surgical mask whereas the fabric in Fig. 6(e) considerably outperforms surgical masks. Conversely, the fabric in Fig. 6(f) performs very poorly; Zangmeister *et al.* writes that this fabric “had visually open weave structures compared to all other fabrics analyzed” (i.e., *g_x_* and *g_y_* are large), suggesting that the fabric is a poor filter from a combination of having a low thread count and thin yarns. The biggest difference we can see between the fabrics in panels (e) and (f) is that (e) has a significantly larger fiber density, as measured through ⟨α⟩ (cf. Table II).

**FIG. 8.**
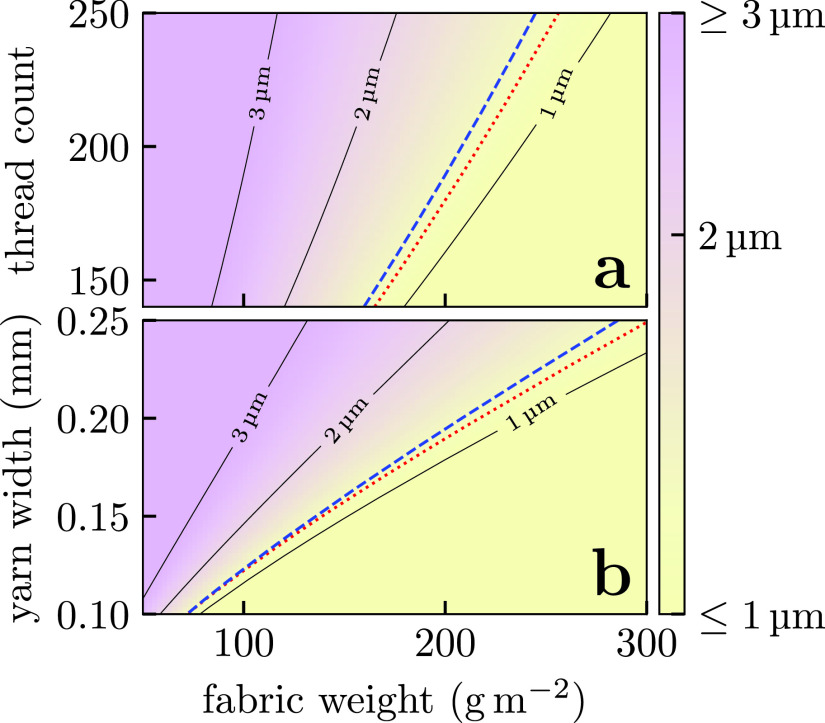
Particle sizes *d_p_* above which woven masks achieve ≥95% filtration. Here, we consider three layers of identical plain-woven cotton fabrics with (a) fixed yarn widths of 0.2 mm and (b) thread counts of 200. For reference, we show lines of surgical mask equivalents (blue dashed line) and where the pressure drop across the mask exceeds the 245 Pa threshold set by the National Institute of Science and Technology (NIST) (red dotted line).^79,80^ We assume identical warp and weft yarns in these calculations. The thread count and fabric weights refer to the properties of individual layers rather than the final multilayered structure.

While our model is clearly approximate, it allows us to explore a much wider range of parameters than is typical of experiments to determine the key parameters for effective masks. In Fig. 8, we show how filtration efficiency is expected to be more strongly influenced by the fabric weight than the thread count or yarn sizes in woven fabrics. The fabric weight is influenced by the thread count, but also by the details of the fabric pattern, the yarn “crimp” [i.e., how meandering the yarn is in Fig. 7(d)] and the structure of the yarns themselves (i.e., how many fibers protrude from the central core). All else being equal, increasing the fabric weight corresponds to an increased ⟨α⟩: this may indicate that the inter-yarn pores are more populated with fibers and gives some crude indication of the fabric's 3d structure. This is broadly consistent with the explanations proposed by Zangmeister *et al.* for their best performing fabrics.

## V. DISCUSSION AND CONCLUSIONS

Masks and face-coverings affect two of the steps in the transmission of a respiratory infection such as COVID-19. These are exhalation from an infected person and inhalation by a susceptible person. Mask effectiveness is not independent of other aspects of transmission, for example, mask efficiency is highest for droplets so large they sediment rapidly. Sedimentation and aerosol dilution play crucial roles at large physical separations and so mask-wearing is not a substitute for physical distancing.

The basic physics of filtration by fibrous filters means that filtering out particles of diameter ≳3 μm is straightforward to achieve in standard fabrics. Moreover, some fabrics are expected to effectively filter ≥95% particles of diameter ∼1 μm, which is comparable to surgical masks; an example is the first woven cotton fabric studied in Ref. 45 and shown in Fig. 6(b). Our model makes austere assumptions, so further experiments would be required to refine the parameter range over which these are equivalent. In particular, the fiber density must be characterized in the inter-yarn pores where most of the air flows through.

For fibers of typical diameters of order O(10 μm), the Stokes number is of order one or more, and so droplets of this size cannot follow the air streamlines faithfully. They then deviate from the path of the air flowing through the mask, and so collide with the fibers and are filtered out. However, filtering out sub-micrometer droplets is much harder as these faithfully track the streamlines of air flowing through the mask. Without introducing electrostatic interactions, which feature in common fabrics only to a very limited extent it is hard to see how to reliably filter out droplets in this size range. The sharp crossover leading to efficient filtration of particles 1 to 3 *μ*m in diameter emerges from an underlying dynamical transition that was first studied in Ref. 77, and so we expect this to be a robust result.

Even masks made from simple cotton fabrics are predicted to reduce transmission of respiratory viruses, unless transmission is dominated by sub-micrometer droplets. As masks are cheap, and wearing a mask is a relatively minor inconvenience compared to contracting SARS-CoV-2, recommending mask use is a simple way to reduce transmission. A simple face covering will never completely eliminate transmission, as some virus-laden droplets will always bypass it. However, unless transmission is dominated by sub-micrometer droplets, mask use should suppress onwards transmission of the virus. To the best of our knowledge, sub-micrometer droplets are highly unlikely to carry significant viral loads.^73,74^

Rather than mandating medical-grade PPE, policy makers could pursue a strategy of improving the quality of cloth masks worn in community settings. Our theoretical model enables the systematic exploration of the mask parameters, which provides a route to optimize mask performance. We have shown that under ideal conditions, cloth masks can be optimized to perform as well as surgical masks. However, the practical performance of any particular mask (cloth or surgical) will crucially depend on the quality of the face seal.^73,82^ Practical guidance on reducing leakage would therefore be required to pursue this strategy. For example, Duncan *et al.*^82^ found that surgical masks sealed via tie straps offered better face seal than ear loops.

The limited data available on face seal suggests the leakage of a *single* mask is typically around ∼25% to 50%,^83,84^ corresponding to effectively ∼5% to 25% when both inhaler and exhaler are masked. Even with this leakage, we would expect a 50% to 75% reduction in exposure to viral particles larger than ≥1 μm under universal masking, or 15% to 50% for sub-micrometer droplets. Note that a reduction in basic reproduction number *R* from $R_{0}=4$ by a conservative 25% would prevent ∼75% of cases during one month of exponential growth assuming a case doubling time of 3.5 days.^85^

Our calculations relied on the standard models of the physics of filtration by fibrous filters. These capture the essential physics, but rely on simple, two-dimensional, models. We have generalized these models to incorporate the polydisperse fiber diameter distributions obtained from SEM experiments, as well as to treat the hierarchical (yarn) structure in woven fabrics in an *ad hoc* fashion. There is scope for future work to look at fully three-dimensional models, models where droplets do not couple to the flow field just at the center of mass, and models for the fiber/droplet interaction.

By focusing on filtration, we have neglected how the mask intervenes with airflow around the mouth and nose, which can significantly change the location and rate of droplet deposition.^86,87^ Xi *et al.*^86^ have found that mask wearing strongly perturbs air flow and hence droplet deposition in the respiratory tract, which implies that the reduction in particles deposited in the respiratory tract will be different from the reduction due to filtration. The authors of Refs. 86 and 87 did not consider the size-dependence of filtration efficiency, so combining these approaches is a potential avenue for future work.

## SUPPLEMENTARY MATERIAL

See the supplementary material for the explicit Kuwabara flow field parameters, details of the Lattice Boltzmann simulations and tests validating the filtration theory, scanning electron microscope images and parameters of fabrics obtained from their analysis, the standard model used for treating diffusion collection efficiency, and the electrostatic potential around cylindrical fibers.

## Data Availability

The code used to do the calculations in this work is available at Ref. 88. The data that support the findings of this study are available from the corresponding author upon reasonable request.
